# A novel CREBBP mutation and its phenotype in a case of Rubinstein–Taybi syndrome

**DOI:** 10.1186/s12920-022-01335-4

**Published:** 2022-08-19

**Authors:** Qian Wang, Cong Wang, Wen Bin Wei, Wei Ning Rong, Xiang Yu Shi

**Affiliations:** 1 Beijing Key Laboratory of Intraocular Tumor Diagnosis and Treatment, Beijing Ophthalmology and Visual Sciences Key Lab, Medical Artificial Intelligence Research and Verification Laboratory of the Ministry of Industry and Information Technology, Beijing Tongren Eye Center, Beijing Tongren Hospital, Capital Medical University, 1 Dong Jiao Min Xiang, Beijing, 100730 China; 2 Ningxia Eye Hospital, People’s Hospital of Ningxia Hui Autonomous Region, Third Clinical Medical College of Ningxia Medical University, Huanghe Road, Jinfeng District, Yinchuan, 750002 Ningxia Hui Autonomous Region China

**Keywords:** Glaucoma, Rubinstein–Taybi syndrome, CREBBP mutation, Phenotype

## Abstract

**Background:**

This study was to report a novel CREBBP mutation and phenotype in a child with Rubinstein–Taybi syndrome.

**Methods:**

Case report of a 9-year-old boy.

**Results:**

We described the patient’s clinical manifestations in detail, and found that in addition to the typical systemic manifestations of the syndrome, the outstanding manifestation of the child was severe intellectual deficiency and prominent ocular abnormalities. Whole-exome sequencing and sanger sequencing were performed on the patient and his parents, a large intragenic deletion, covering the exon 1 region and part of the intron 1 region of the TRAP1 gene, and the entire region from intron 27 to exon 30 of the CREBBP gene (chr16:3745393-3783894) was identified on the patient. This mutation affected the CREBBP histone acetyltransferase (HAT) domain.

**Conclusions:**

This findings in our patient add to the spectrum of genetic variants described in Rubinstein–Taybi syndrome and present a RSTS patient with various ocular anomalies including early onset glaucoma.

## Introduction

Rubinstein–Taybi syndrome (RSTS), first described in 1963 by Rubinstein and Taybi, is a rare congenital neurodevelopment disorder characterized by facial dysmorphism, mental deficiency, growth retardation, and a variety of systemic abnormalities [1, 2]. Fetal growth rate is generally unaffected in RSTS and the abnormalities are often noted at birth and during early infancy. The height, weight and head circumference rapidly drop below the fifth percentile in the first few months of life. Adult patients are usually short in stature and become overweight relative to height during puberty and adulthood [3–5]. The diagnosis is usually based on the clinical criteria, including the presence of mental retardation associated with three major symptoms, such as broad thumbs or first toes, thick eyebrows or long eyelash, and columella below alae nasi [6].

The inheritance pattern of RSTS is autosomal dominant with an estimated prevalence of 1 in 100,000–125,000 live births [7, 8]. The majority of cases (~ 99%) occur sporadically de novo [9] although a few familial cases have been reported [10–12]. In most cases, parents of RSTS patients are not affected. When parents are clinically unaffected, sibs are still presumed to be at increased risk of RSTS due to the presence of mild phenotypes in heterozygous parents or parental somatic cells and/or germ line chimeras. Individuals with RSTS rarely reproduce. Pathogenic variants of two highly evolutionarily conserved genes are associated with the incidence of RSTS, CREBBP and EP300. 50–60% of RSTS patients are caused by mutations of CREBBP [13], 80% of which are associated with pathogenic sequence variants and 20% with deletion of variable sizes [14]. Pathogenic variants of EP300 were found in 8–10% of individuals with RSTS, predominantly frameshift type, while deletional type rarely reported [15]. However, the genetic basis of the remaining 30% of RSTS patients remains unclear [16].

In Chinese RSTS patients, a higher percentage of microcephaly, micrognathia, polydactyly and syndactyly but a lower percentage in urogenital anomalies has been noted [17, 18]. Yu and colleagues reported two relative “hot spot” regions in CREBBP gene for truncated and deletion variants in a Chinese cohort (18 kids) [18]. One is a 461-nt long region (codons 1931 and 2086) in exon 31 of CREBBP gene, and another region locates in exon 2 at the 5’ end of the CREBBP [18]. In the past, most cases of RSTS were discovered and reported by pediatricians, and there were few reports on the ocular phenotype of RSTS [19]. In this manuscript, we describe a novel CREBBP mutation in a Chinese RSTS patient first diagnosed with congenital glaucoma to broaden the genetic variant spectrum of this rare disease.

## Methods

The patient and his parents were recruited in accordance with the principles of the Declaration of Helsinki. The study protocol was approved by the Medical Ethics Committee of the Beijing Tongren Hospital and written informed consent was obtained from the Legal guardians of the patients (parents) for participation in this study and to publish study finding.

### Clinical examinations

The ophthalmological examinations included measurement of visual acuity, tonometry, slit lamp-assisted biomicroscopy of the anterior segment of the eye. We conducted ocular biometry for the determination of axial length using optical low-coherence reflectometry (Lenstar 900 Optical Biometer, Haag-Streit; 3098 Koeniz, Switzerland). Applying a non-mydriatic fundus camera (CR6-45NM; Canon Inc., Õsta, Tokyo, Japan), we obtained 45° fundus photographs.

### Whole exome sequencing

Samples of peripheral venous blood (5 ml) were collected from patient and his parents for genomic DNA extraction using a QIAmp DNA Mini Blood Kit (Qiagen, Hilden, Germany). To reveal the disease causing mutation in this family, whole exome sequencing approach was performed on this family. Briefly, the libraries for whole exome sequence were established from the DNA samples using an exon capture kit (SureSelect ver. 6 + UTR, Agilent Technologies), according to the manufacturer’s instructions. The exons were sequenced as 100-basepairs paired-end reads by an Illumina HiSeq2500 (Illumina). The original sequencing data were processed by Illumina Basecalling Software 1.7 and compared with NCBI HUMAN genome DNA reference sequence (NCBI Build 37.1). Single nucleotide variation (SNV) information is analyzed using SOAP software (http://soap.Genomics.org.cn). The information related to Inserts and deletions (Indel) is analysed using BWA software (http://bio-bwa.sourceforge.net/) to obtain all the mutations occurring in the DNA sequences of the sample. The common variants (MAF > 1%) that appear in the database (DB135) are filtered out, and then those that have no effect on the structure and function of the protein are filtered out. Candidate pathogenic gene variants were obtained by filtration.

### In silico analysis

The possible effects of the amino acid changes on the function was predicted by using the PolyPhen-2 (http://genetics.bwh.harvard.edu/pph2/), the Sorting Tolerant from Intolerant (SIFT) algorithm (http://sift.jcvi.org/www/SIFT_enst_submit.html), and the MutationTaster (http://www.mutationtaster.org/). Mutations were classified as clinically unclear when at least one of the four predictions had a benign outcome or when there was insufficient evidence of pathogenicity. When all the predicted results were pathogenic, the variation was classified as possible pathogenic variation combined with other evidence. Frameshift variation, nonsense variation and variation with experimental evidence of protein function loss are classified as pathogenic variation. Sanger sequencing was performed for intrafamilial cosegregation analysis. The allele frequencies of the mutations in the normal population were viewed in 1000 Genome (http://browser.1000genomes.org/index.html), EVS (http://evs.gs.washington.edu/EVS/) and EXAC (http://exac.broadinstitute.org/) databases.

## Results

### Clinical evaluation

**Fig. 1:**
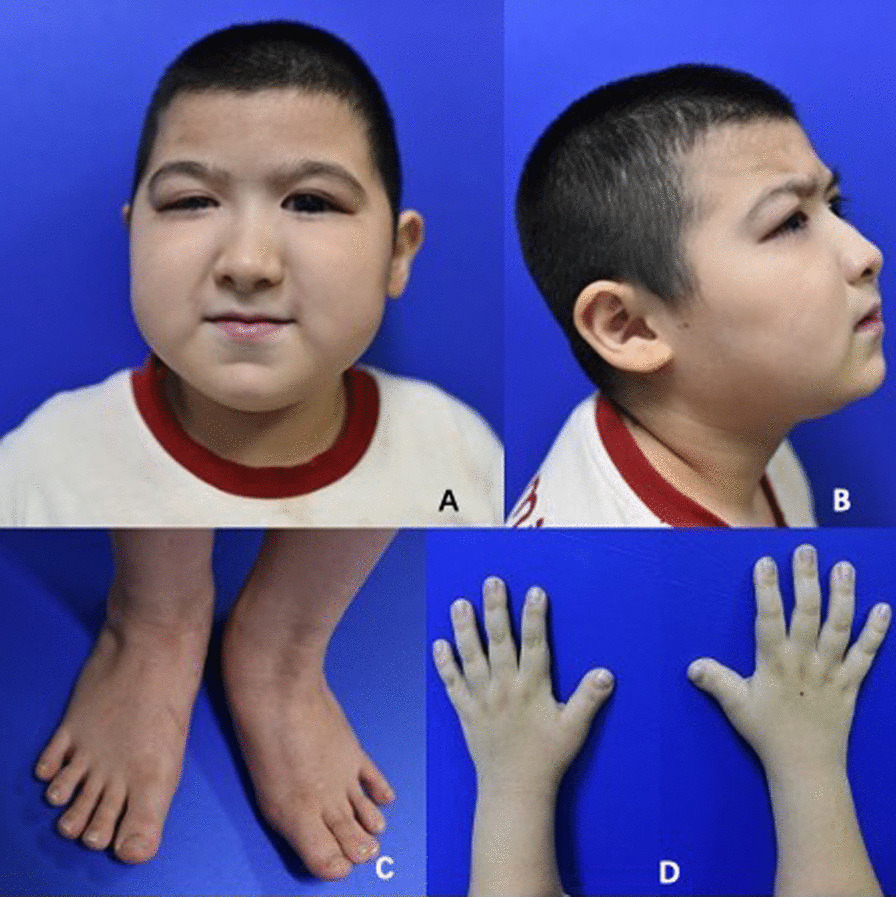
The patient had the characteristic facial dysmorphism and facial grimacing seen in RSTS, including microcephaly, high arched eyebrows, low-set ears, low and thick columella, thin upper lip, a protuberant lower lip (**A**, **B**), broad thumbs, fetal finger pads, and broad great toes (**C**, **D**)

The 9-year-old Chinese boy had low normal height, truncal obesity and low normal skull circumference (On examination, his weight was 65 kg, height was 124 cm, and head circumference was 49 cm). He had severe mental retardation (IQ estimated to be 10–20, according to Zhang et al. 2005) [20], and his psychomotor development was delayed. Shortly after the child was born, the parents found that the development of the child was slower than that of other normal children, and the time of raising his head, crawling, and standing was significantly delayed, and he was unable to perform fine operations or walked steadily. The child's language development is also obviously delayed. Until now, he can only understand some simple daily sentences, and can only speak some simple words, and cannot communicate normally. He had the characteristic facial dysmorphism and facial grimacing seen in RSTS. His distinctive features included microcephaly, high arched eyebrows, low-set ears, low and thick columella, thin upper lip, a protuberant lower lip, grimacing smile with almost closed eyes on smiling. He also had broad thumbs, persistent fetal finger pads, and broad great toes (Fig. 1).

**Fig. 2:**
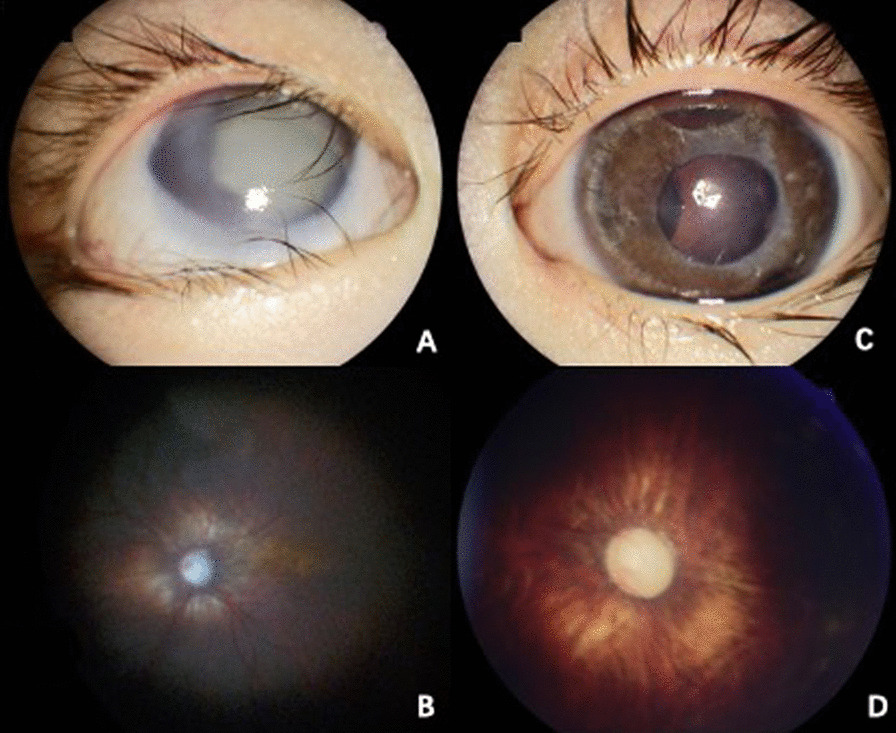
In his right eye, ectopia lentis was noted, and he had a cataract in the lens, corneal clouding, iris coloboma, and no light reflex (**A**). Fundus photography showed optic disc pale and choroidal atrophy in the right eye (**B**). In the left eye, he had relatively clear cornea, iris atrophy with peripheral incision in the superior (**C**), pale optic disc and choroidal atrophy (**D**)

The patient was diagnosed as congenital glaucoma and underwent trabeculotomy twice at the age of 1 and 2-year-old, respectively. Combined with Latanoprost, Brinzolamide and Timolol Maleate eye drops, the intraocular pressure of both eyes have been normal since the surgery until 5 months ago, after going through the surgery of correction of lower eyelid inversion combined with ptosis. When admitted to the hospital, the patient can’t cooperate with visual acuity or other ocular examination. Examination of the eyes under general anesthesia showed a horizontal corneal diameter of 13 mm in the right eye and 15 mm in the left eye. Schiotz tonometry showed an intraocular pressure of 40.08 mm Hg in the right eye and 14.57 mm Hg in the left eye. The axial lengths were 29.7 mm in the right eye and 30.8 mm in the left eye. In his right eye, ectopia lentis was noted, and he had a cataract in the lens, corneal clouding, iris coloboma, and no light reflex. In the left eye, he had relatively clear cornea, iris atrophy with peripheral incision in the superior, pale optic disc and choroidal atrophy (Fig. 2). The patient underwent vitrectomy, lens cutting, and internal transscleral ciliary body photocoagulation in his right eye. The intraocular pressure of the child was maintained at a normal level after the surgery.

This patient was born to a healthy nonconsanguineous Chinese couple. Anterior segment and fundus examination was unremarkable in this couple. The family history was otherwise noncontributory.

### Mutation analysis and in silico analysis

**Fig. 3:**
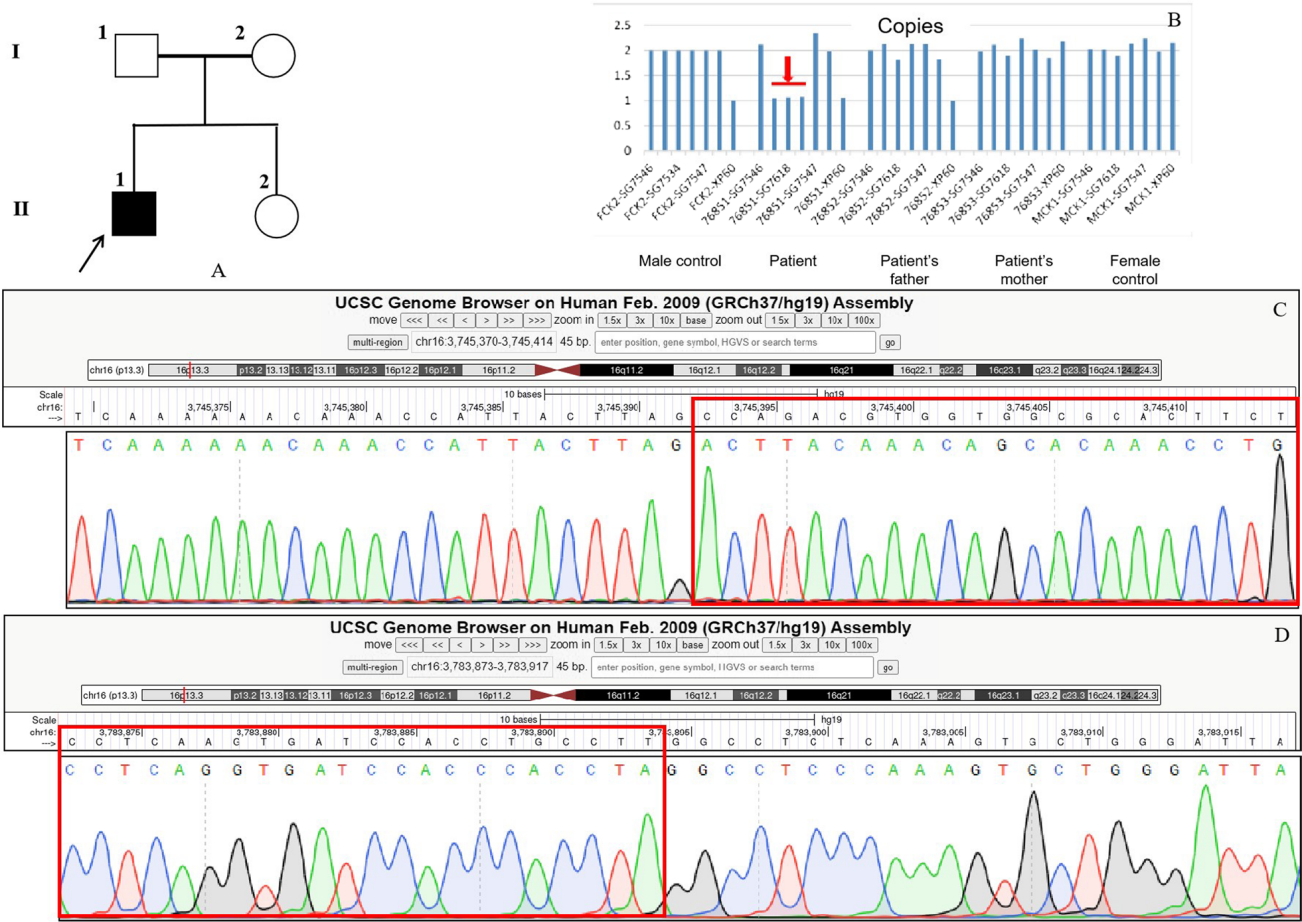
Pedigree of affected family (A). Whole exome sequencing analysis identified a large heterozygous copy number deletion on CREBBP gene, from exon 29 to exon 31 (chr16 3778026-3781885) on the patient, this mutation was de novo and not found on his parents by sanger sequencing (**B**). Long PCR and Sanger sequencing showed that the heterozygous copy number deletion region chr16:3745393-3783894 covers the exon 1 region and part of the intron 1 region of the TRAP1 gene, and the entire region from intron 27 to exon 30 of the CREBBP gene (**C**, **D**)

Chromosome analysis in peripheral lymphocytes showed a normal male karyotype (46,XY) at a banding resolution of 400 bands per haploid genome. Whole exome sequencing analysis was performed on the patient, a large heterozygous copy number deletion on CREBBP gene, from exon 29 to exon 31 (chr16 3778026-3781885) was identified on the patient, this mutation was de novo and not found on his parents by sanger sequencing (Fig. 3). We used long PCR and Sanger sequencing to determine the location of the breakpoint on the patient, and found that the upstream breakpoint position of the heterozygous copy number deletion is chr16:3745392, and the downstream breakpoint position is chr16:3783895. It is confirmed that the size of the heterozygous copy number deletion is 38503 bp, and a sequence of nearly 300 bp has been inserted at the breakpoint. It might be caused when DNA double-strand break damage is repaired (Fig. 3). This heterozygous copy number deletion region chr16:3745393-3783894 covers the exon 1 region and part of the intron 1 region of the TRAP1 gene, and the entire region from intron 27 to exon 30 of the CREBBP gene. In general, this pathogenic heterozygous deletion is the causative mutation for the disease phenotype in this family.

## Discussion

Rubinstein–Taybi syndrome is a rare genetic disorder characterized by postnatal growth retardation, moderate to severe mental retardation, and multiple characteristic malformations. Despite its prominent facial features, broad thumbs and hallux, the diagnosis of RSTS is sometimes challenging due to the high variability of phenotypes and genotypes [21]. Whole exome sequencing is a reliable and rapid diagnostic tool for suspected genetic diseases and has been strongly applied in medical practice [18].

To date, 500 pathogenic variants have been described in CREBBP [9], and a pathogenic variant in CREBBP is identified in 50–60% of all RSTS cases. It has been reported that the mutation spectrum included 80.2% of point mutations, of which truncating mutations accounted for 55.2% of all point mutations, followed by large rearrangements (18.8%), missense mutations (16.8%), and splice mutations (9.2%) [9]. CREBBP has no real hotspot mutation sites, and its mutation spectrum is distributed along all of the 31 exons [9]. However, studies have found some recurrent mutations, such as about 52% of missense mutations are located in the location of histone acetyltransferase (HAT domain) [9]. In up to 30% of patients with suggestive clinical manifestations, the genetic etiology of RSTS remains unknown [22], possibly due to unrecognized genetic variants.

By now, no convincing genotype–phenotype correlation in RSTS has yet been established [23], though recent studies produced insight into the role of the CREBBP and EP300 genes in neural cell and brain development, regulating precursor cell migration and neuronal plasticity [24–27]. The syndrome has been subdivided into type 1 associated with the CREBBP mutation spectrum (RSTS1; OMIM#180849) and type 2 associated with the EP300 mutation spectrum (RSTS2; OMIM#613684). There are different phenotypes of RSTS1 [28]. The classic phenotype, which caused by deletions or truncating mutations of the CREBBP gene, is characterized by intellectual disability, broad thumbs, and characteristic facial dysmorphism [29]. A very severe phenotype of RSTS1, also known as the chromosome 16p 13.3 contiguous deletion syndrome, caused by large deletions including the CREBBP gene and the 3’ adjacent genes, viz., DNASE1 and TRAP1 [28], always exhibits severe mental retardation, life-threatening infections and systemic complications, and other classic features [30]. However, more and more evidence showed that no significant correlation shown between the phenotype and the mutation type and location or deletion size for either CREBBP or EP300 genes in RSTS patients [15, 31–33].

In this study, we report the deletion of exon 27 to exon 30 of the CREBBP gene and the 3’ adjacent genes, TRAP1, in one patient molecularly confirming the diagnosis of RSTS. To best of our knowledge, this mutation has not been published previously. We described the patient’s clinical manifestations in detail, and found that in addition to the typical systemic manifestations of the syndrome, such as intellectual disability and facial dysmorphism, the outstanding manifestation of the child was ocular abnormalities (infantile glaucoma, ptosis and epicanthus) and significantly lower IQ (10–20). The child’s IQ was significantly lower than most other reports (average IQ was reported between 35 and 50) [34], who can only understand simple, daily sentences and single words, has no language or only a few words, and usually walks unsteadily. Since no observable genotype–phenotype relationship has been observed for other mutations in RSTS, this mutation is less likely to lead to a specific clinical RSTS subtype.

Intragenic deletion was report in 10%-23% of the CREBBP-mutated patients [33] and the reported CREBBP deletions were spread along the gene (Table 1). The deletions vary in size and genomic location, some involving the whole CREBBP gene and its flanking regions, while others only involving an intragenic portion [33, 35–37]. There are currently no reports in which ethnic groups or regions of the population with CREBBP gene deletions are more common [31–33, 35, 38–44], while it seems that deletions more frequently involve the HAT region [32]. In Chinese patients, CREBBP intragenic deletions account for 11–37% of CREBBP pathogenic genes [17, 18]. Ye and colleagues reports a relative “hot spot” in CREBBP for deletion, locating in exon 2 at the 5’ end of the CREBBP [18]. Half of the patients carried deletions of five exons or more [31]. Although not confirmed in other populations [33], the association between severe phenotypes and deletion of CREBBP and adjacent genes has been reported [30]. In addition, researchers did not find an association of larger deletions with disease severity [31, 45]. No correlation was observed between the location of the affected exons and the phenotype [31, 32]. However, previous studies have reported a worse cognitive phenotype in patient carriers of mutations that affect the CREBBP HAT domain [33], which also seen in our case. CREBBP is highly conserved, with 95% homology between the human and murine genes [46]. The reported mutational spectrum of CREBBP, including deletions, point mutations, and large rearrangements, distributed throughout the 31 coding exons. However, the highly conserved HAT region is believed to be particularly important for the RSTS phenotype. A large study including 93 patients with RSTS found that almost all the truncating mutations lead to premature termination of the protein before or within the HAT domain (corresponding to exons 19–30) and there was a clustering of single amino acid changes in the HAT region, indicating the importance of HAT activity in the phenotype [13, 44]. In this patient, three exons (exons 27–30) of CREBBP are deleted. This is predicted to disrupt HAT activity and supports the importance of the HAT domain in causing the phenotype of RSTS.

**Table 1:** CREBBP deletions reported in previous studies

No	CREBBP deletion	Databases (Decipher, public HGMD, LOVD) and references
1	Ex1del	HGMD (Breuning et al.[33])
2	Ex2del	LOVD (Breuning et al. [33])
3	Ex1-2del	LOVD (Breuning et al. [33])
4	Ex1-3del	Choi et al. [32]
5	Ex1-19del	Roelfsema et al. [29]
6	Ex1-31del	LOVD (Mogensen et al. [34], Bentivegna et al. [35])
7	Ex2-3del	Cross et al. [27]
8	Ex3-31del	Pérez-Grijalba et al. [26]
9	Ex4-16del	HGMD (Bentivegna et al. [35], Rusconi et al. [28])
10	Ex6-13del	Cross et al. [27]
11	Ex6-31del	HGMD (Rusconi et al. [28], Choi et al. [32])
12	Ex7-8del	Cross et al. [27]
13	Ex9-14del	Cross et al. [27]
14	Ex12-31del	Rusconi et al. [28]
15	Ex17-28del	Cross et al. [27]
16	Ex17-31del	HGMD (López et al. [36], Choi et al. [32])
17	Ex20-31del	Elalaoui SC, et al. [37]
18	Ex21del	Pérez-Grijalba et al. [26]
19	Ex22-23del	HGMD (Bentivegna et al. [35])
20	Ex24-28del	Cross et al. [27]
21	Ex24-31del	Pérez-Grijalba et al. [26]
22	Ex26-30del	Pérez-Grijalba et al. [26]
23	Ex27-28del	Cross et al. [27]
24	Ex27-29del	Cross et al. [27]
25	Ex27-30del	This study
26	Ex29-30del	Pérez-Grijalba et al. [26]
27	Ex29-31del TRAP1	Lai et al. [38], Rusconi et al. [28]
28	Ex31del	HGMD (Bentivegna et al. [35], Rusconi et al. [28])

In our patient, another affected gene was the TRAP1 gene encoding tumor necrosis factor receptor-associated protein 1. As a mitochondrial ATP-binding protein, TRAP1 gene participants in maintaining mitochondrial function [47]. TRAP1 also acts as a molecular chaperone and is a heat shock protein 90-related protein [48]. However, the TRAP1 gene has not been associated with human disease [28] and whether the deletion of TRAP1 is associated with a more severe phenotype is also inconclusive. Rusconi et al. reported a slight increase in growth retardation in patients carrying deletions involving not only CREBBP but also other genes like TRAP1[33]. However, the presentations of two patients described by Stef et al. [37] and one patient reported by Lai et al. [44] did not support the description of a severe RSTS phenotype in patients with a large deletion involving CREBBP and contiguous genes, such as DNASE1 and TRAP1.

In addition, deletion of HAT domain of CREBBP has been reported to causing ocular abnormality [7, 31]. Various ocular features have been reported in RSTS with a high occurrence of 80% [19], particularly refractive errors (56%) and strabismus with subsequent risk of amblyopia (71%) [49]. These usually respond well to interventions. Patients with RSTS also have a higher frequency of lacrimal duct obstructions (38–47%), which may require surgical intervention. Other serious ocular abnormalities were also described, such as corneal abnormalities (19.7%, such as megalocornea without glaucoma, opacities, keretoglobus, sclerocorenea), congenital glaucoma (26.5%), congenital cataract (12.8%), and coloboma (33.3%) [49]. Peri-ocular findings adding to the characteristic facial appearance has been described: ptosis, downward slanted palpebral fissures, hypertelorism and epicanthus [49]. The presence of childhood glaucoma in the present patients corresponds to the ocular spectrum of RSTS. Brei and colleagues found 32 glaucoma and 25 corneal opacities in 614 patients with RSTS, and they concluded that the incidence of glaucoma in RSTS exceeds that of the general population and is often congenital or develops in infancy [50]. Since congenital glaucoma is a preventable cause of blindness and early treatment is critical, it is especially important to request a detailed ophthalmological examination to rule out ocular complications after diagnosis [34]. It is recommended that all children with RSTS should undergo a detailed ophthalmologic examination by a pediatric ophthalmologist shortly after diagnosis, or at 6 months of age in patients with RSTS diagnosed in the neonatal period. If an eye problem is suspected, referral to an ophthalmologist should be made as soon as possible. Depending on the examination results, regular ophthalmological visits should be performed every 12 months or less [19].

Although whole exome sequencing has the advantage of high efficiency and convenience in screening pathogenic genes, the importance of traditional sanger sequencing cannot be ignored. For example, in our research, we find a large deletion mutation of exon 29 to 31 on CREBBP gene, after verification by Sanger sequencing, it was finally confirmed to be the mutation covers the exon 1 region and part of the intron 1 region of the TRAP1 gene, and the entire region from intron 27 to exon 30 of the CREBBP gene.

## Conclusions

The genetic variant found in this case was reported in this work for the first time, hence, contributing to the RSTS molecular knowledge and expanding the CREBBP genetic variant repertoire of this complex disorder. Meanwhile, this study further suggests the importance of ophthalmic examination and follow-up in patients with RSTS. Timely and correct treatment for congenital glaucoma and other ophthalmic diseases is helpful to improve the quality of life of children.

## Data Availability

The datasets generated during the current study are available in the DDBJ BioSample repository, the sample of the patient can be obtained from the web link: https://ddbj.nig.ac.jp/resource/biosample/SAMD00469867; the sample of the patient’s father can be obtained from the web link: https://ddbj.nig.ac.jp/resource/biosample/SAMD00469868; the sample of the patient’s mother can be obtained from the web link: https://ddbj.nig.ac.jp/resource/biosample/SAMD00469869
